# The occurrence, types, reasons, and mitigation strategies of defensive medicine among physicians: a scoping review

**DOI:** 10.1186/s12913-022-08194-w

**Published:** 2022-06-20

**Authors:** Edris Kakemam, Morteza Arab-Zozani, Pouran Raeissi, Ahmed Hassan Albelbeisi

**Affiliations:** 1 Clinical Research Development Unit of Tabriz Valiasr Hospital, Tabriz University of Medical Sciences, Tabriz, Iran; 2 Social Determinants of Health Research Center, Birjand University of Medical Sciences, Birjand, Iran; 3 Department of Health Services Management, School of Health Management and Information Sciences, Iran University of Medical Sciences, Tehran, Iran; 4 Medical Services Directorate, Gaza Strip, Palestine

## Abstract

**Background:**

Defensive Medicine (DM) concept refers to all medical care provided by physicians without increasing the benefits to the patient, the primary purpose of which is to prevent the risk of litigation. Although several studies have been published investigating the occurrence of DM around the world, no review conducted on DM among physicians. Therefore, this study aims to summarize and map the available evidence on occurrence, types of behaviors, and reasons for practicing of DM among physicians and possible solutions and strategies to reduce DM in the literature.

**Methods:**

This is a scoping review in which we searched Web of Science, Scopus, and PubMed in December 2021. Our target was original studies of any type that included data on DM among physicians between 2000 and 2021. We followed the JBI guideline for conducting a scoping review and for increasing the rigor of the study. First, the percentage was used to summarize the occurrence of DM, and then, findings related to types of behaviors and reasons for practicing DM and mitigation strategies were analyzed inductively in NVivo 10 in three stages.

**Results:**

Twenty-seven studies were included in the review. The overall occurrence of DM practice ranged from 6.7 to 99.8%. Two types of DM behaviors including assurance and avoidance behaviors have been identified. The common reasons for practicing DM were categorized into four themes, patient-related reasons, physician-related reasons, organization-related reasons, and society-related reasons. The main strategies to prevent or reduce DM are structured training and education, restoring physician-patient relationships, reform of the health system, and reform of the liability system.

**Conclusions:**

The vast majority of research studies were conducted in high-income countries, and studies are needed to measure this phenomenon and its consequences in depth in low- and middle-income countries. Various solutions and strategies are needed to reduce defensive behaviors such as structured training and education, restoring physician-patient relationships, reforming the health system, and reforming the liability system.

**Keywords:**

Defensive medicine, Defensive practice, Medical malpractice, Physicians, Scoping review

**Supplementary Information:**

The online version contains supplementary material available at 10.1186/s12913-022-08194-w.

## Background

The concept of Defensive Medicine (DM) appeared in the United States between 1974 and 1978 and then extended all over the world [1, 2]. Principally, the DM concept refers to all medical care provided by physicians without increasing benefits to the patient, the primary purpose of which is to prevent the risks of litigation [2–4].

The DM actions could be negative or positive, depending on the condition. The negative actions include avoiding comorbidity patients or high-risk health services; thus, patients are excluded from treatment and hospitalization, and the positive actions include unnecessary medical procedures or investigations, and prescribing unnecessary drugs [2, 5, 6].

Previous studies hypothesized that the practice of DM is associated with many causes, such as increased costs of medical error insurance premiums, and patients’ bias to sue for missed or delayed treatment services [2, 7]. Generally, DM reflects the behavior of healthcare providers that aims to prevent malpractice from administrative, legal, criminal, and ethical penalties [8]. The consequences of DM such as drug overuse and low-quality care, are emerging as an important concern in modern health policy and practice [9]. Although the cost of DM is unclear, a previous study showed that the majority of physicians believe that DM increases the cost of health care services [10]. In the USA, DM costs are estimated to be between $46 billion and $300 billion annually, which is about 3.0% of national health spending, a study of physicians caring for elderly patients estimated that DM costs ranged from 8.0 to 20.0% of total health costs [10, 11]. In Italy, it is estimated that DM costs about 10.0% of national health spending [12].

DM is popular among physicians who specialize in critical surgery, including General Surgery, Orthopedics, Gynecology, and Neurosurgery [13–15]. The concept of DM is gaining attention among different countries around the world [4–6, 12, 13, 16].

The occurrence of DM varies from one country to another and from one medical specialty to another, high rates were estimated 99.8% in Iran [17], 98.0% in Japan [18], 88.0% in the United States [19], 71.8 in Sudan [20], 72.0% in Turkey [21], 60.0% in Italy [12], and between 52.0 and 62.0% in Israel [22, 23]. Based on the performed preliminary searches in databases: Scopus, Cochrane, and PubMed, several studies have been published investigating the occurrence of DM around the world and no review conducted on DM among physicians.

Several studies have been written on DM. Knowing the status of DM research in the literature is valuable for understanding current knowledge, designing evidence-based interventions, and conducting further future research. Therefore, this study aims to summarize and map the available evidence on occurrence, types of behaviors, and reasons for practicing DM among physicians and possible solutions and strategies to reduce DM in the literature.

## Methods

The latest JBI guidance for scoping reviews was used to conduct the current scoping review [24]. We chose the scoping review method due to the exploratory and descriptive nature of the study objectives [25]. The basic descriptive was used to summarize the occurrence of DM. Then, the qualitative description was used to identify DM types, reasons for practicing DM, possible solutions, and strategies to reduce DM.

### Step 1. Identifying the research question

A scoping review generally starts with one or more questions. Hence, in this scoping review, we sought to answer the following questions:

Overarching question:

What knowledge is available about DM among physicians in the literature?

Sub-questions:

1. What is the proportion of occurrence of DM in studies?
2. What kinds of behaviors of DM are available among physicians?
3. What are the reasons for practicing DM?
4. What are possible solutions and strategies to reduce DM in studies?

### Step 2. Search strategy

Based on the performed preliminary searches in databases: Scopus, Cochrane, and PubMed, no review was conducted on DM among physicians. After that, keywords from the studies were identified by the research team. Then, with the help of a librarian was developed a search strategy. A literature search of the Web of Science, Scopus, and PubMed databases was conducted in December 2021 using the terms “defensive medicine”, “defensive practice”, “defensive” “Physicians”, “Surgeons”, “doctor”, “specialist”, and “General Practitioners”. In the peer-reviewed literature search, the keywords were combined using the Boolean term “AND” and “OR” in all the electronic databases explored. A full list of searches carried out can be found in Supplementary file 1. The references used in the journal articles included in the final analysis were also screened to identify relevant studies.

### Step 3. Inclusion criteria

Our target was empirical/original studies that included data on DM among physicians between 2000 and 2021. All studies that report at least one of the items of occurrence, types of behaviors, reasons, or solution or strategies to reduce it among physicians from any specialty (General Practitioners, Surgeons, and specialists) in a healthcare setting were included. Also, all original studies written in English and published in peer-review journals were considered. We excluded studies if the full text was not available, and the results did not match with aims of the study. The studies conducted among medical students and residents excluded as well.

### Step 4. Evidence screening and selection

**Fig. 1:**
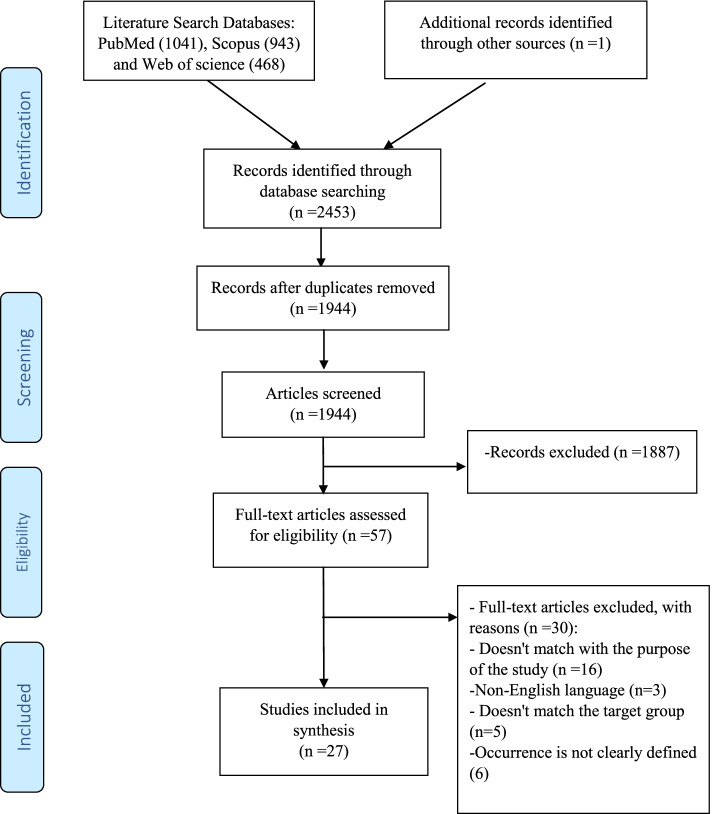
Flow diagram of studies retrieval

All references retrieved through the initial search were saved in an EndNote® library (9.3) and reviewed for relevance. Additional publications were identified by reviewing the reference lists of relevant papers. The studies were selected based on the inclusion and exclusion criteria. Studies selection was started with a review of both titles and abstracts using the inclusion criteria. Following abstract review, full texts were accessed for final screening and data extraction. This process was conducted by two reviewers (EK and AHB), and any disagreements resolved by either consensus or with a third reviewer (PR). The study selection process was presented in Fig. 1. Critical appraisal or risk of bias assessment is commonly not conducted in scoping reviews because the aim is to map the available evidence rather than provide a synthesized and clinically meaningful answer to a question [24].

### Step 5. Data extraction

We created a data extraction form to collect data. Details of articles including publication details [e.g., first author and year of publication]; country, study population, and study design [e.g., sampling methods and sample size]; occurrence of DM, reasons for practicing DM, and possible solutions and strategies to reduce DM were extracted and charted using Microsoft Excel. Then, papers were independently confirmed by two researchers (AHA and EK) before concluding inclusion in a review. When there were disagreements between reviewers in the paper review and selection process, a third reviewer (MAZ) was involved to make a final decision.

### Step 6. Data analysis

In the first step, the basic descriptive (percentage) was used to summarize the occurrence of DM. Next, our data charting and exploration were guided by Thomas and Harden’s three-stage approach to qualitative data synthesis [26]. The results sections from all records were imported into NVivo 10, analyzed inductively and coded line-by-line by [EK and AHA], using codes discussed and agreed with [MAZ]. We then grouped related codes to create new ‘top line’ codes to describe the groupings and used these ‘top line’ codes to create analytical themes. We finally tabulated the records to make the distributions of themes across the review clear.

## Results

### Study selection

A total of 2453 studies were retrieved articles from various databases by using the search strategy elaborated earlier. Out of 2453 studies, 1944 studies were unique. During the initial period of screening, the 1944 studies were checked based on their title and abstract. Thereafter, 1887 studies were excluded and the remaining 57 studies were full-text evaluated based on the inclusion and exclusion criteria. After that, 27 studies were selected and involved in the review analysis (Fig.1).

### The characteristics of the selected studies

Table 1 showed the characteristics of the 27 studies involved in the review [2, 12, 14, 15, 17–23, 27–42]. The included studies were published between 2002 and 2020 and distributed as follows: six in the USA [14, 19, 29, 32, 37, 42], four in Italy [12, 30, 34, 35], three in Israel [22, 23, 28], two studies per country, China [15, 33],Turkey [2, 21], Netherlands [38, 41], UK [27, 31],one study per country, Iran [17], Sudan [20], Japan [18]. Three studies were conducted as an international, the first in Canada, South Africa, and the USA [36], the second in nine countries in the Middle East [40], the third in 74 countries [39].

**Table 1:** Characteristics of the included studies and frequencies of occurrence of defensive medicine

Authors/Publication year	Country	Study population	Study design	Sampling methods	Sample size (n)	Occurrence (%)
Passmore et al./2002 [27]	England	Psychiatrists	Postal survey	Census	154	75.0
Studdert et al./2005 [14]	USA	Emergency medicine, General surgery, Orthopedic surgery, Neurosurgery, Obstetrics/ Gynecology, and Radiology	Mail survey	Randomly	824	93.0
Hiyama et al./2006 [18]	Japan	Gastroenterologists	Survey	Randomly	131	98.0
Asher et al./ 2012 [28]	Israel	Internal medicine, Pediatrics, General surgery, Family medicine, Obstetrics/ gynecology, Orthopedic surgery, Cardiology, and Neurosurgery	Survey	Randomly	889	60.0
Nahed et al./2012 [29]	USA	Neurosurgeons	National online survey	NS	1028	36.0–72.0
Elli et al./ 2013 [30]	Italy	Gastroenterologists	Survey	NS	107	94.0
Ortashi et al./2013 [31]	UK	Medicine, Surgery, Obstetrics /Gynecology, Pediatrics	Survey	Convenience	204	78.0
Sathiyakumar et al. /2013 [32]	USA	Orthopedic (trauma and non-trauma)	National Survey	Randomly	1214	84.0–86.0
He et al./ 2014 [33]	China	Internal Medicine, Surgery, Obstetrics/Gynecology, Pediatrics	Survey	Randomly	504	80.6
Moosazadeh et al./ 2014 [17]	Iran	General practitioners	Survey	Census	423	79.2–99.8
Solaroglu et al./2014 [21]	Turkey	Neurosurgeons	Survey	NS	404	72.0
Ramella et al./ 2015 [34]	Italy	Radiation oncology	Survey	NS	361	75.0
Reisch et al./2015 [19]	USA	Breast pathologists	National online survey	NS	252	88.0
Ali et al./2016 [20]	Sudan	Obstetrics/Gynecology	National Survey	NS	117	71.8.0
Panella et al./2016 [35]	Italy	General Surgeons, Anesthesiologists, Internists, Pediatricians, Psychiatrics, Emergency, Radiologists, Cardiologists, Urologists, Pathologists, Neurologists, Rehabilitation Doctors	National online survey	Randomly	1313	59.7
Silberstein et al./2016 [23]	Israel	Plastic and Aesthetic Surgery	Prospective survey	NS	78	51.3
Yan et al./2016 [36]	Canada, South Africa, USA	Neurosurgery	Online survey	NS	1142	64.5–89.1
Din et al./2017 [37]	USA	Neurosurgery	Online survey	NS	1026	84.6–89.2
Panella et al./2017 [12]	Italy	Physicians	National survey	Randomly	1313	59.8
Reuveni et al./2017 [22]	Israel	Psychiatrists	Survey	NS	213	62.1
Yan et al./2017 [38]	Netherlands	Neurosurgeons	National online survey	NS	45	6.7–64.4
Tebano et al./2018 [39]	74 countries	Infectious diseases and Clinical microbiology doctors	International online survey	NS	830	76.0–85.0
Zhu et al./2018 [15]	China	Obstetrics/Gynecology	National online Survey	NS	1486	62.9
Al-Atram et al./2018 [40]	9 Middle Eastern countries	Psychiatrists	Survey	NS	92	30.0
Renkema et al./2019 [41]	Netherlands	Anesthesiology, Colon, stomach and liver diseases, Gynecology, Internal medicine, Neurology, and Surgery	Electronic Survey	NS	214	42.0–89.0
Borgan et al./2020 [42]	USA	Internal Medicine Residents	Online survey	Convenience	49	40.0–91.3
Calikoglu et al./2020 [2]	Turkey	Anesthesia, Obstetrics/Gynecology, Ear Nose Throat Physician, General Surgery, Urology, Eye Diseases, Orthopedics, Cardiovascular, Surgery, Neurosurgery, Plastic surgery, Thoracic, and Pediatric	Survey	All practicing physicians	190	94.2

*USA* United States of America, *UK* United Kingdom, *NS* Not Stated, *UAE* Unite Arab Emirates

### Participants

The 27 selected studies included 14,603 physicians from various specialties. The sample size ranged from 45 to 1486. Seven studies involved more than 1000 physicians [12, 15, 29, 32, 35–37]. The studies conducted among different disciplines and distributed as follows: five among neurosurgeons [21, 29, 36–38], three among psychiatrists [22, 27, 40], two studies for each specialty, Gastroenterologists [18, 30], Obstetrics/Gynecology [15, 20], nine studies among General practitioners or more than one specialty [2, 12, 14, 17, 28, 31, 33, 35, 41], and one study for each specialty, Orthopedic [32], Radiation oncology [34], Breast pathologists [19], Plastic and Aesthetic Surgery [23], Infectious diseases and Clinical microbiology doctors [39], Internal Medicine [42].

### Occurrence of defensive medicine

Table 1 reports the occurrence of DM in 27 studies. The occurrence of DM practice ranged from 6.7% in a study conducted among neurosurgeons in the Netherlands to 99.8% among general practitioners’ study in Iran [17, 38].

### The occurrence of defensive medicine by specialty

Regarding neurosurgeons, the highest occurrence of DM was recorded in the United States at 89.2% and the lowest recorded among Dutch neurosurgeons at 6.7% [37, 38]. Regarding Psychiatrists, the highest occurrence was recorded in England at 75.0% and the lowest recorded among psychiatrists in nine countries in the Middle East at 30.0% [27, 40]. Regarding Gastroenterologists, the highest occurrence was recorded in Japan at 98.0% and the lowest recorded in Italy at 30.0% [18, 30]. Regarding Obstetrics/Gynecology, the highest occurrence was recorded in Sudan at 71.8% and the lowest recorded in China at 62.9% [15, 20]. Regarding General practitioners or more than one specialty, the highest occurrence was recorded in Iran at 99.8% and the lowest recorded in the Netherland at 42.0% [17, 41].

### The occurrence of defensive medicine in countries

The vast majority of research studies were conducted in one country, except three research studies conducted as international studies [36, 39, 40], as well as in high-income countries, except studies conducted in Sudan, Turkey, Iran, and some countries included in the study conducted in 74 countries, and the study conducted in 9 Middle Eastern countries [2, 17, 20, 21, 39, 40].

In high-income countries, DM practice in the USA ranged from 36.0% among neurosurgeons to 93.0% among various specialties [14, 29]. In Italy, DM occurrence ranged from 59.7% among various specialties to 94.0% among gastroenterologists [30, 35]. In Israel, DM occurrence ranged from 51.3% among plastic and aesthetic surgery to 62.1% among psychiatrists [22, 23]. In China, DM occurrence ranged from 62.9% among obstetrics/gynecology to 80.6% among various specialties [15, 33]. In the Netherlands, the occurrence ranged from 6.7% among neurosurgeons to 89.0% among various specialties [38, 41]. In the UK, the occurrence ranged from 75.0% among psychiatrists to 78.0% among various specialties [27, 31].

In low- and middle-income countries with deficiencies in the capacity and preparedness of health care systems [43]. In Turkey, the occurrence of DM practice ranged from 72.0% among neurosurgeons to 94.2% among various specialties [2, 21]. In Iran, 99.8% among general practitioners study, in Sudan, 71.8% among Obstetrics/Gynecology [17, 20].

### Types of defensive medicine behaviors

In Table 2 we categorized DM behaviors into assurance and avoidance themes. Although DM is generally considered as a negative behavior. Assurance behaviors are usually not detrimental to patients. While avoidance behaviors are detrimental for patients. The most common and most consistently reported types of assurance defensive behaviors in the included studies were prescribed unnecessary services, unnecessary referral patients to other specialties and hospitals, suggesting and performing unnecessary invasive procedures, and spending more time with patients and their families. The most common and most consistently reported types of avoidance defensive behaviors in the included studies were avoiding conducting effective high-risk procedures/interventions, and use non-invasive protocols, avoiding admitting and care high-risk patients, performing unnecessary intervention surgery, and avoiding switching to oral treatments. More details are provided in Supplementary file 2.

**Table 2:** Characteristics and types of defensive medicine behaviors reported in the included studies

Main Themes	Sub-themes	Samples codes
**Assurance behaviors (not detrimental to patients)**	• Prescribe unnecessary services	• Prescribing unnecessary medication or antibiotics’ • Request unnecessary laboratory tests and investigations • Request unnecessary imaging • Calling unnecessary examinations and consultations • Ordering more consultations on probable complications • Selecting the more extremist diagnosis for borderline cases
	• Unnecessary referral cases to other specialties and hospitals	• Refer patients to other specialists unnecessarily • Send patients to emergency room, in unnecessary conditions
	• Unnecessary cases admission and hospitalization	• Hospitalized patient who can be treated as an outpatient
	• Suggest and perform unnecessary invasive procedures	• Ordering unnecessary biopsies • Ordering unnecessary endoscopies
	• Spend more time with patients and their family	• Describe medical procedures to patients in more details • Request additional reviews • Increases follow-up • Initiates communication with family • More patients’ observations than required
**Avoidance behaviors (detrimental to patients)**	• Avoid applying effective high-risk procedures / interventions, and use non-invasive protocols	• Ceasing high-risk procedures • Avoid treatment protocols or guidelines with high complication
	• Avoiding to admit and care high-risk patients	• Withdraw from practice entirely and retire • Avoid patients with complex medical problems
	• Performed unnecessary intervention surgery	• Perform cardio-pulmonary resuscitations and intubations for poor prognosis patients • Caesarean section without indications • Excising skin lesions that are not suspected of being malignant
	• Avoiding switching to oral treatments	• Avoid stop parenteral drugs

### Reasons for practice behaviors of defensive medicine

We summarized the main reasons for practicing DM behaviors mentioned in 27 included studies into four main themes (Table 3 and Supplementary 2). The most common reasons for practicing DM were patient-related reasons, physician-related reasons, organization-related reasons and, society-related reasons.

**Table 3:** Reasons for practicing defensive medicine and strategies to reduce defensive medicine reported in the included studies

Reasons for practicing DM		Possible solutions and strategies to reduce DM	
Main themes	Codes	Main themes	Codes
**Patient-related reasons**	• Increasing number of lawsuits against physicians • Physicians’ self-perceived threats from patients • Past disputes with patients • Avoid potential conflict with patients • Patient pressure factors	**Structured training and education**	• Improve Physicians training educating and about appropriate care in clinical surroundings • Implementation of awareness programs about the DM phenomenon • Establish and disseminate clinical protocols or guidelines targeting widespread DM actions • Support the regular use of evidence-based medicine and structured care • More training in problem-solving techniques • Health curriculum should specifically address litigation issues
**Organization-related reasons**	• Increasing malpractice premiums • Decreasing provider reimbursement • Inadequate medical and or organizational procedures • Inadequate malpractice and liability coverage • Inadequate hospital support for liability issues • Inadequate legislation protecting doctors	**Physician–patient relationship**	• Restore trust in physician -patient relationships • Innovate harmony and alliance between physician and patient • More communication with patients and their families • Induct social workers to participate in managing the conflict between physicians and patients • Promoting the ethical values of physicians
**Physician-related reasons**	• Solo practice • Previous experience of complaints and legal claim for themselves and colleagues • A perceived legal risk • Fear and concerns over medical liability • Physicians Low-income • Concerns about financial and possible legal consequences • Lack of self-confidence • Lack of specialized knowledge • The weekly activity volume • Ineffective physician–patient relationship • Legal protection • Feared compromising their professional reputation and or career	**Reform of the health system**	• Redistribution of the health procedures between various healthcare professionals, and enhance multidisciplinary collaborations • A comprehensive examination of main factors and the expenditure on DM, and a better understanding of the current shortages in the healthcare system • Establish clinical records management • Better use of the risk management techniques • Establish clinical auditing system and health debriefing • Physician reward system reform • Forming a committee to study malpractice cases to avoid recurrence • Performing a compensation procedure for a patient who has suffered a medical injury
**Society-related reasons**	• Concerns about media attention • Believe in working in a blame-free culture • A general negative context surrounding negligence claims against physicians	**Reform the liability system**	• Ways of complaints and inquiry should be upgraded. • Introduce complaints committee in hospitals • Filtration of cases at an early stage to prevent the court as much as possible • Establish alternatives to the existing litigation system • Establishment of health courts and specialized courts with trained judges in the field of health care

### Possible solutions and strategies to reduce defensive medicine in the included studies

Studies have reported many possible solutions and strategies to reduce DM behaviors. These solutions were also grouped into four main themes (Table 3 and Supplementary 2). The most common reported solutions and strategies to reduce defensive behaviors in the included studies were structured training and education, restoring physician-patient relationships, reform of the health system, and reform of the liability system.

## Discussion

The concept of DM is gaining attention among different countries around the world. Although several studies have been published investigating the occurrence of DM around the world, no review summarized the occurrence of DM. The latest JBI guidance for scoping reviews was used to conduct the current review. The basic descriptive was used to summarize the occurrence of DM. Then, the qualitative description was used to identify DM types, reasons for practicing DM, possible solutions, and strategies to reduce DM. A total of 27 research papers (14,603 physicians) conducted between 2000 and 2021 were selected and involved in the review analysis. The occurrence of DM practice ranged from 6.7% in a study conducted among neurosurgeons in the Netherlands to 99.8% among general practitioners study in Iran [17, 38]. There is evidence that DM is not only practiced by physicians [44–46]. Previous studies showed that 32.0–53.0% of midwives claimed to have changed their practices in a preventive manner [45, 47]. A recent systematic review demonstrated that many DM behaviors were practiced, 41.5% of midwives and nurses reported improving documentation, 7.6% consent gathering for all procedures, and 23.0% said they handle high-risk care cases less [48]. A study conducted among medical students showed that 94.0% practice DM behaviors [49].

There were differences between specialties and countries in the spread of DM. The vast majority of research studies conducted in one country, except three research studies conducted as international studies [36, 39, 40], as well as in high-income countries, except studies conducted in Sudan, Turkey, Iran, and some countries included in the study conducted in 74 countries, and the study conducted in 9 Middle Eastern countries [2, 17, 20, 21, 39, 40]. Studies are needed to deep measure the phenomenon and its consequences in low and middle income countries.

The most commonly reported types of assurance defensive behaviors in the included studies were prescribed unnecessary services such as medications, laboratory tests, and medical imaging, unnecessary referral patients to other specialties and hospitals such as sending patients to the emergency room in unnecessary conditions, suggest and perform unnecessary invasive procedures such as ordering unnecessary biopsies and endoscopies and spend more time with patients and their family such as increases follow-up more than required. Previous studies have shown that health care providers may write incorrect medical notes to protect themselves and organizations [45]. These behaviors can lead to waste financial and non-financial resources, increase health care costs, affect the quality of the health care system, and affect health care accessibility and availability [6, 50–52].

The most commonly reported types of avoidance defensive behaviors in the included studies were avoiding conducting effective high-risk procedures/interventions, and use non-invasive protocols, avoiding admitting and care high-risk patients, performed unnecessary intervention surgery. These behaviors can lead to adverse effects on patient health outcomes and healthcare providers, for example, some studies reported that Physicians Withdraw from practice entirely and retire [21, 36].

The most common reasons for practicing DM were patient-related reasons such as the increasing number of lawsuits against physicians, and potential conflict with patients. Many studies showed the need to restore trust in physician-patient relationships, innovate harmony and alliance between physician and patient, and more communication with patients and their families [12, 14, 20, 28, 33]. In addition, physician-related reasons such as solo practice, physicians’ low income, previous experience of complaints, and legal claims for themselves and colleagues, the included studies demonstrated the need for redistribution of the health procedures between various healthcare professionals, enhance multidisciplinary collaborations, promote ethical values of physicians, physician reward system reform, and improve physicians training educating and about appropriate care in clinical surroundings [2, 12, 14, 18, 33–36, 41].

Furthermore, organization-related reasons and, society-related reasons such as inadequate malpractice and liability coverage, inadequate hospital support for liability issues, inadequate legislation protecting doctors, increasing malpractice premiums, concerns over medical liability, concerns about possible legal consequences, feared compromising their professional reputation and or career, concerns about media attention, and a general negative context surrounding negligence claims against physicians.

Based on the included studies, the possible solutions to reduce the organizations and society reasons mentioned in the included studies were improving physicians’ training, educating about appropriate care in clinical surroundings, establishing and disseminating clinical protocols or guidelines targeting widespread DM actions, supporting the regular use of evidence-based medicine and structured care, induct social workers to participate in managing the conflict between physicians and patients, establish clinical records management, establish clinical auditing system and health debriefing, forming a committee to study malpractice cases to avoid recurrence, ways of complaints and inquiry should be upgraded, establish alternatives to the existing litigation system, and establishment of health courts and specialized courts with trained judges in the field of health care [2, 12, 14, 15, 17, 18, 20–22, 29, 30, 32, 35, 38–40].

## Limitations of study

There are many potential limitations to this scoping review. First, a literature search was conducted in the three major electronic databases, Scopus, Pubmed, and Web of sciences, but no other databases were searched, as was the ‘gray’ literature. Therefore, additional relevant studies might have been missed. Second, in line with the scope review methodology, the risk of bias was not assessed in the included studies. Although systemic heterogeneity was noted in the included studies, based on quality assessment studies were not included/excluded, as a systematic review would be necessary. Therefore, caution is advised when drawing conclusions based on these studies’ combined data. Third, published papers on DM have focused on a small portion of medical professionals, including neurosurgeons, psychiatrists, obstetricians, and gynecologists. These studies cannot reflect the practice of DM in other disciplines especially general practitioners.

## Conclusion

The current scoping review has summarized the published research papers on the occurrence, types of behaviors, and reasons for practicing of DM among physicians and possible solutions and strategies to reduce DM in countries around the world. The results of the study showed that DM is a common behavior among physicians. Such that it’s overall occurrence varied from 6.7 to 99.8% in different countries. The vast majority of research studies were conducted in high-income countries, and studies are needed to measure this phenomenon and its consequences in depth in low- and middle-income countries. All studies reported two types of DM (assurance and avoidance behavior). There are various reasons such as patient-related, physician-related, organization-related and, society-related reasons for practicing DM behaviors that need further exploration. However, various solutions and strategies are needed to reduce defensive behaviors such as structured training and education, restoring physician-patient relationships, reforming the health system, and reforming the liability system.

## Supplementary Information

**Additional file 1: Supplementary file 1.** Full list of Search strategy in Three Databases.

**Additional file 2.**

## Data Availability

All relevant data are with the article and the attached Supplementary information.

## Abbreviation

DM: Defensive Medicine
