# Supplementary material for: The occurrence, types, reasons, and mitigation strategies of defensive medicine among physicians: a scoping review
Source: BMC Health Serv Res. 2022 Jun 20;22:800. doi: 10.1186/s12913-022-08194-w (PMC9210603; doi:10.1186/s12913-022-08194-w)
Supplement: Supplementary file 1 — Additional file 1: Supplementary file 1. Full list of Search strategy in Three Databases. [file 12913_2022_8194_MOESM1_ESM.docx]

Supplementary file 1: Full list of Search strategy in Three Databases

| Database | Search strategy | Results |
| --- | --- | --- |
| PubMed | (((defensive medicine[MeSH Terms]) OR (defensive practice[Title/Abstract])) OR ((defensive) AND (medicine OR practice))) AND (((((Physicians[MeSH Terms]) OR (General Practitioners[MeSH Terms])) OR (doctor[Title/Abstract])) OR (Surgeons[MeSH Terms]) ) OR (specialist[Title/Abstract])) | 1041 |
| Scopus | ( TITLE-ABS-KEY ( "defensive medicine"  OR  "defensive practice*" )  AND  TITLE-ABS-KEY ( physician*  OR  "General Practitioner*"  OR  doctor*  OR  surgeon*  OR  specialist* ) ) | 943 |
| WOS | TOPIC: ("defensive medicine"  OR  "defensive practice*") AND TOPIC: (physician*  OR  "General Practitioners*"  OR  doctor*  OR  surgeon*  OR  specialist*)  Timespan: All years. Indexes: SCI-EXPANDED, SSCI, A&HCI, ESCI. | 468 |
| Total= |  | 2452 |
| Duplicate |  | 509 |
| Screen |  | 1943 |
