# Supplementary material for: The occurrence, types, reasons, and mitigation strategies of defensive medicine among physicians: a scoping review
Source: BMC Health Serv Res. 2022 Jun 20;22:800. doi: 10.1186/s12913-022-08194-w (PMC9210603; doi:10.1186/s12913-022-08194-w)
Supplement: Supplementary file 2 — Additional file 2. [file 12913_2022_8194_MOESM2_ESM.docx]

**Supplementary file**

**Reasons for practicing DM actions and possible solutions included in the studies**

| **Study** | **Aim/Purpose** | **DM Actions’ (Assurance and Avoidance behaviors*)** | **Reasons for practicing DM** | **Possible solutions and strategies to reduce DM** |
| --- | --- | --- | --- | --- |
| 1. Passmore et al/ 2002. | - To examine the extent of defensiveness among psychiatrists and to examine the relationship between defensiveness and seniority, as well as the effect of previous experiences on the level of defensiveness | - Admit patients to hospital - More patients’ observations than needed - Recurrent write in the patient's files - Dictating | - A Previous experience of complaints or Colleague experience of complaints - Concerns about media attention - Previous critical incident - A previous legal case against the colleague or against self | - More structured training - Methods for complaints and investigations should be improved. |
| 1. Studdert et al/2005. | - To study the prevalence and characteristics of defensive medicine among physicians practicing in high-liability specialties | - More medical tests or investigations (CT, MRI, US, Radiography, Angiography, Biopsy) - Refer patients (not necessary) to more specialist doctors - More prescribed drugs - Unnecessary invasive procedures’ - Avoid particular interventions* - Avoid high risk patients* | - lacked confidence in their liability coverage - The perception of premium burdens as extreme - Solo practice | - Increase efforts to reduce DM procedures and these efforts should focus on educating patients and Physicians about suitable care in clinical circumstances - Develop and disseminate clinical protocols or guidelines targeting common DM practices - Reducing the financial and psychological vulnerability of individual physicians in high-risk specialties for system liability shocks. |
| 1. Hiyama et al/2006 | - To clarify the prevalence of defensive medicine and the specific defensive medicine practices among gastroenterologists in Japan | - Order more tests than medically indicated - Prescribe more drugs like antibiotics than medically indicated - Refer patients to other specialists unnecessarily - Suggest invasive procedures like biopsy to confirm diagnosis - Avoid certain procedures or interventions* - Avoid caring for high-risk patients* | - Previous sued experience - The increase in the number of litigations - Mass media reports of medical malpractice | - Increase efforts to reduce DM procedures and these efforts should focus on educating patients and gastroenterologists about suitable care in clinical circumstances - Develop and disseminate clinical protocols or guidelines targeting common DM practices |
| 1. Asher et al/ 2012. | - To measure the extent and characteristics of defensive medicine among Israeli board certified expert physicians from high- and low-risk specialties. | - Perform more tests (blood, chest x-ray, brain CT) - Refer patients to other specialists unnecessarily - Admit more patients unnecessarily - Suggest invasive procedures like biopsy to confirm diagnosis - Prescribe more medications - Perform life resuscitations and intubations for poor prognosis patients - Less frank with their patients. - Avoid certain procedures or interventions* - Stop practicing or eliminate high-risk procedures* | - A potential threat for a medical lawsuit - A previously been sued | - Regain trust in physician-patient relationships. |
| 1. Nahed et al/2012. | - To determine beliefs and self-reported practices of neurosurgeons to determine how the perception of malpractice risk affects routine practice | - Ordering additional, imaging studies - Laboratory tests - Referring patients to consultants - Prescribing medications - Eliminated high-risk procedures* | - Increasing malpractice premiums - A perceived legal risk - Concerns over medical liability - Patient as a potential lawsuit - Decreasing provider reimbursement | - Create alternatives to the current tort litigation system - Introduce health courts, specialized courts with judges trained in healthcare, which are meant to limit the number of frivolous lawsuits |
| 1. Elli et al/ 2013. | - To clarify the impact of defensive medicine on gastroenterological practices in Lombardy. | - Defensively requested procedures - Endoscopies - Abdominal ultrasonography scans - Abdominal CT, MRI - Referring patients to consultants | - Increasing number of lawsuits against physicians - Increasing Insurance premiums for specialists - Mass media inviting patients to make malpractice claims | - Create alternatives to the current tort litigation system |
| 7. Ortashi et al/2013. | - To assess the prevalence of the practice of defensive medicine in the UK among hospital doctors and the factors affecting it. | - Ordering unnecessary tests - Unnecessary referral to other specialties - Prescribing un-necessary medication - Performing unnecessary procedures - Refuse to treat high risk patients* - Avoid high risks procedures* | - Believe in working in a blame-free culture | - NA |
| 1. Sathiyakumar et al /2013. | - To survey the prevalence of defensive medicine in orthopaedic trauma surgery | - Radiography, CT, MRI, US - Referrals - laboratory tests - Biopsies - Admissions - Reduce the number of high-risk patients and procedures* | - liability concerns | - Policies aimed at managing liability risk may be useful in containing practices - Medical liability reform |
| 1. He et al/ 2014. | - To explore the doctor-patient relationship, defensive medicine and overprescription in Chinese public hospitals | - Prescribing unnecessary diagnostic tests, drugs and therapeutic interventions. | - Physicians' self-perceived threats from patients - Past disputes with patients - Physicians Low-income - Avoid potential conflict with patients - Retain essential documents in the face of lawsuits | - Reform doctor-patient relationship - Reforming the physician remuneration scheme - Introduce compulsory medical malpractice insurance for health professionals - Recruit social workers to share in conflict management between Physicians and patients |
| 1. Moosazadeh et al/ 2014. | - To determine frequency of positive and negative defensive medicine behaviors and their underlying factors among general practitioners | - Prescribing unnecessary Para-clinical orders, treatment, drugs - Unnecessary referral to other specialties - Avoiding to provide high-risk procedures for patents* - Avoiding to admit high-risk patents | - Being informed of lawsuits against colleagues - Consequences of complaints - Financial consequences - lack of self-confidence - lack of specialized knowledge | - Some of DM actions is learnt at health colleges which again demands for a new perspective in health learning. - The need for creating customized protocols and clinical guidelines |
| 1. Solaroglu et al/2014. | - To investigate the characteristics of defensive medicine, its reasons, and the extent to which it is practiced in the Turkish health care system | - Ordering additional imaging studies - Ordering additional laboratory tests - Prescribe unnecessary medications - Referring patients to consultants - Suggest an invasive procedure to confirm diagnosis like biopsy - Avoid practicing or eliminate high-risk procedures or interventions - Avoiding high-risk surgery* | - A potential threat in terms of a medical lawsuit - The courts can distinguish malpractice from complications - Protect themselves from legal risks | - Introduce special malpractice courts - Creating a forum to discuss malpractice cases (medical and law communities) - Making compensation process for a patient who faced medical injury |
| 1. Ramella et al/ 2015. | - To explore frequency and nature of defensive practices among radiation oncologists | - Ordering further imaging studies, Laboratory tests - Referring patients to consultants - Prescribing additional medication - Order hospitalization in a patient who can be managed as an outpatient - Suggest invasive procedures against professional judgement - Avoid caring for high-risk patients - Avoid conducting certain high-risk procedures/interventions even if to the patient’s benefit | - Fear of a claim for damages or of legal litigation in the event of complications/adverse events - Previous experience of legal problems - The current public opinion towards doctors | - Implement programmes aiming at improving awareness of the phenomenon and to increase good clinical practice. |
| 1. Reisch et al/2015. | - The extent to which concerns about medical malpractice influence assurance behaviors of pathologists interpreting breast specimens | - Ordering additional immunohistochemistry tests - Recommending additional surgical tests - Request additional reviews - choosing the more severe diagnosis for borderline cases | - Concerns over malpractice liability - Exposure sued for medical malpractice | NA |
| 1. Ali et al/2016 [23] | - To assess the concept of defensive medicine (in term of knowledge and prevalence) and to determine any experience of medical litigations | - Arranging un-necessary refer - Ordering unnecessary investigations - Prescribed un-necessary medication - Lack of hospitals’ clinical guidelines - Avoiding high risk procedures* - Refused manage high risk patient* - Performed un-necessary surgery* | - Avoid litigation and criticism - Concerns of possible legal consequences - Experience of litigation - To protect themselves from blame and   litigation and some fears | - Create trust, rapport, and alliance between Physician and patient - Strategies to reduce medical litigation against Physicians |
| 1. Panella et al/2016. | - To study the condition of being second victim as a possible determinants of defensive medicine among Italian hospital physicians. | - Prescribe unnecessary laboratory test - Prescribe unnecessary diagnostic tests or procedures - Prescribe specialist consultations - Prescribe unnecessary medications - Transfer patients to emergency room, in unnecessary circumstances - Transfer patients to hospital in unnecessary circumstances - Avoid performing high-risk procedures* - Avoid treating high-risk patients* | - Physicians’ experience of being a second victim after an adverse event - The weekly activity volume | - Redistributing the activity level among the clinical teams - Discuss the law of tort - Use of evidence-based clinical guidelines |
| 1. Silberstein et al/2016. | - To examine the extents of intended and unintended Defensive medicine among members of the Israeli Society of Plastic and Aesthetic Surgery and identified risk factors for Defensive medicine | - Referring the patient for unnecessary consultations - Ordering unnecessary tests - Excising skin lesions that were not suspicious for malignancy - Performing unnecessary administrative procedures | - Exposure to previous litigation - Exposure to previous malpractice claims | NA |
| 1. Yan et al/2016 [15] | - To compare defensive medicine practices in Canada, South Africa, and the United States | - Ordering additional imaging - laboratory tests - Consults - Additional referrals - Avoiding high risk procedures* - Withdraw from practice entirely and retire* | - Ongoing medical liability crisis - View their patients as potential lawsuits - Exposure to claims in past 3 years | - The need for comprehensive analysis of predictors and costs of defensive behavior could lead to a better understanding of the inefficiencies that currently exist in the health care system |
| 1. Din et al/2017. | - To compare defensive practices of U.S. spine and non-spine neurosurgeons in the context of state medical liability risk | - Ordering additional laboratory tests - Unnecessary referrals - Ordering additional medications - Ordering additional imaging - Discontinuing high-risk procedures* | - Malpractice claims - Insurance malpractice coverage was inadequate - A liability crisis existed - Patients as potential lawsuits | - NA |
| 1. Panella et al/2017. | - To identify the prevalence of the practice of defensive medicine among Italian hospital physicians, its costs and the reasons for practising defensive medicine and possible solutions to reduce the practice of defensive medicine | - Order more diagnostic tests or procedures than medically indicated - Order more lab tests than medically indicated - Refer to specialists in unnecessary circumstances - Prescribe more medications than medically indicated - Refer patients to emergency room in unnecessary circumstances - Admit/transfer patients to hospital in unnecessary circumstances - Avoid conducting effective high-risk procedures/ interventions - Avoid caring for high-risk patients | - A general negative context surrounding negligence claims against physicians - Inadequate legislation protecting doctors - Fear of medical lawsuits - Concerns about medical injury compensation for malpractice - Feared compromising their professional reputation and or career - Ineffective physician–patient relationship - Mass media and public attitudes towards medical practices - Inadequate medical and or organizational procedures - Inadequate liability insurance - Inadequate hospital support for liability issues | - Systematic use of evidence-based medicine and organized care (clinical pathways, protocols - More attentive, daily communication with patients and families - Implementation of a more systematic education to continuously improve physicians’ knowledge and skills - Better management of clinical records - Strengthening physicians’ ethical values - More attentive, daily communication with other colleagues in multidisciplinary team collaborations - Use of better risk management procedures - Implementation of systematic clinical auditing and medical debriefing |
| 1. Reuveni et al/2017. | - To assess the scope of DP practiced by psychiatrists and to understand whether awareness of DP correlated with defensive behaviours | - Increases follow-up - Initiates contact with family - Consults senior psychiatrist - Refers to another professional - Prescribes medication without indication | - Exposure malpractice claims | - Changes in the physicians and patients, perspective and behaviours - Giving more information to the public regarding the recommended care and relevant diagnostic and treatment options - Developing and applying clinical practice guidelines - Reforms in the liability and compensation systems available |
| 1. Yan et al/2017. | - To explore perceived liability burdens and self-reported defensive behaviors among neurosurgeons in the Netherlands and compare their practices with their non- European counterparts | - Ordered laboratory tests - Referred patients - Prescribed medication - Suggested a procedure   Ordered imaging | - Previous legal complaints - Previous sued   Previous civil court claims | - Introduce alternatives to civil court claims such as a hospital’s complaints committee |
| 1. Tebano et al/2018. | - To investigate fear of legal claims and defensive behaviors among specialists in infectious diseases and clinical microbiology and to identify associated demographic and professional characteristics | - Prescribing antibiotics even if not really necessary - Prescribing unnecessarily broad-spectrum antibiotics or combinations of agents - Prescribing an antibiotic because of a patient’s perceived expectation. - Avoiding switching to oral treatments* | - Fear of legal liability | - Introduce guidelines and sharing decisions through teamwork |
| 1. Zhu et al/2018. | - To determine prevalence, patterns and risk factors of defensive medicine | - Prescription unnecessary examinations/ tests/recipes/consultations - Arrangements for unnecessary hospital administration/surgeries - Caesarean section without indications | - Medical disputes, lawsuits, loss of a lawsuit experienced by colleagues | - Develop and disseminate clinical protocols or guidelines targeting common DM practices |
| 1. Al-Atram et al/2018. | - To examine the prevalence of defensive practice among psychiatrists in Middle Eastern countries | - Admitted patients to hospital when the patient’s condition could be managed in an outpatient - Placed patient on a higher level of observation than warranted by patient condition - Written in patient records specific remarks such as “not suicidal” which you would not have if you were not worried about legal/media/ disciplinary consequences - Dictated letters more than necessary for managing patient’s illness | - Previous experience of complaints - Colleagues’ previous experience of complaints - Previous legal claim - Colleagues’ previous legal claim - Previous critical incident - Concerns about media interest | - More systematic problem‑based training, - Proper guidelines for practice insurance and more clarity - Transparency in the investigation and handling of medicolegal issues may help - Improve better patient care |
| 1. Renkema et al/2019. | - This study investigated whether the attitudes of physicians towards justified and unjustified litigation, and their perception of patient pressure in demanding care, influence their use of defensive medical behaviours | - Prescribing unnecessary medication, - Ordering tests that are not clinically indicated, - Carrying out unnecessary procedures - Making unnecessary referrals. - Refusing to treat high-risk patients* - Refusing to treat patients with complex medical problems* - Refusing to treat patients from whom one expects an accusation* | - Patient pressure factors - Negative litigation attitudes - Unjustified litigation with stressful long-lasting litigation procedures - Negative media attention | - Filtering out unjustified cases at an early stage to prevent the court - Find a way to prevent physicians from go through unjustified litigation proceedings - Health curricula should specifically address litigation attitudes and how to deal with patient pressures. |
| 1. Borgan et al/2020. | - To examine the prevalence and patterns of defensive medical practices among internal medicine residents | - Order more tests than medically indicated - Prescribe more medication than clinically indicated - Consult specialists more often than clinically indicated | - Risk of being sued - Patients as a potential lawsuit - Medico-legal risk | - NA |
| 1. Calikoglu et al/2020. | - To evaluate the defensive medicine knowledge, attitudes, and behaviors of physicians working in the surgical departments of a Turkish university hospital | - Order extra tests - Hospitalize patients for social indications - Prescribed un-necessary medication - More consultations - More imaging techniques - Use non-invasive protocols instead of interventional treatment protocols* - Avoid treatment protocols with high complication* - Avoid patients with complex medical problems* - Avoid patients who are likely to sue | - Avoid legal problems - legal protection - Increased number of malpractice lawsuits | - More training needs - Efforts ought to be made to keep Physician’s anxiety and risk perception in balance |
